# Causal role of immune cells in Hashimoto’s thyroiditis: Mendelian randomization study

**DOI:** 10.3389/fendo.2024.1352616

**Published:** 2024-05-13

**Authors:** Zhendan Zhao, Yuehua Gao, Xiaoqing Pei, Wenhao Wang, Huawei Zhang

**Affiliations:** Department of Ultrasound, Shandong Provincial Hospital Affiliated to Shandong First Medical University, Jinan, China

**Keywords:** immunity, Hashimoto’s thyroiditis, Mendelian randomization, causal inference, thyroid

## Abstract

**Objectives:**

Hashimoto’s thyroiditis (HT) is a common autoimmune disease whose etiology involves a complex interplay between genetics and environment. Previous studies have demonstrated an association between immune cells and HT. However, the casual relationship was not clear. We aimed to explore the causal associations between signatures of immune cells and HT.

**Methods:**

In this study, bidirectional two-sample Mendelian randomization (MR) analysis was conducted to investigate the potential causal relationship between 731 immune cell signatures and HT by using genome-wide association study (GWAS) data. Heterogeneity and horizontal pleiotropy were detected through extensive sensitivity analyses.

**Results:**

The increased levels of six immune phenotypes were observed to be causally associated with increased risk of HT P < 0.01, which were CD3 on CM CD8br, CD3 on CD39+ secreting Treg, HLA DR on CD33dim HLA DR+ CD11b−, CD3 on CD4 Treg, CD62L− plasmacytoid DC %DC, and CD3 on CD45RA+ CD4+. In addition, the levels of FSC-A on HLA DR+ T cell and CD62L on monocyte were associated with disease risk of HT P < 0.01. In addition, HT also had causal effects on CD3 on CM CD8br, CCR2 on monocyte, CD25 on CD39+ resting Treg, and CCR2 on CD62L+ myeloid DC P < 0.05.

**Conclusions:**

In this study, we demonstrated the genetic connection between immune cell traits and HT, thereby providing guidance and direction for future treatment and clinical research.

## 1 Introduction

As one of the most common autoimmune disorders in the world, Hashimoto’s thyroiditis (HT) is characterized by lymphocyte infiltration and thyroid autoantibodies such as against thyroid peroxidase (anti-TPO) or thyroglobulin (anti-TG), which eventually leads to thyroid fibrosis and hypothyroidism (1, 2). According to statistics, there are 0.3–1.5 cases of HT per 1,000 people worldwide, with a male-to-female ratio of 1:7–10 (3). HT is the most common cause of hypothyroidism in iodine-sufficient areas (4). In the current state of science, there are few, if any, effective treatments available for people with HT in addition to thyroid hormone replacement (5). Thus, a better understanding of pathogenesis of HT is vital for the development of more effective treatments.

A growing number of studies have revealed the complex relationships between immune system and HT (6). Laboratory studies demonstrated that Treg cell deficiency was associated with infiltration of the thyroid gland, which could lead to thyroid cell apoptosis and hypothyroidism (7, 8). In addition, Breg cells appears to be involved in HT progression, although its mechanism of action is not yet fully understood (9, 10). In addition, several polymorphisms of cytokine genes, such as IL1 and IL17, were reported to be associated with the production of anti-TPO, which suggests that these cytokines participated in HT progression (11, 12). Although the close relationship between HT and immune system was identified by numerous observation studies, whether this association was causal still remains unknown.

Because genetic variants are randomly allocated at conception, the two-sample Mendelian randomization (MR) method can be used to evaluate the potential causal effect between an exposure and an outcome based on genetic variants, which reduces the effect of confounding factors and conquers reverse causality (13, 14). To further explore the causal relationship between immune cell signature and HT, a bidirectional two-sample MR analysis was conducted to examine the influence of immune cells on HT.

## 2 Methods

### 2.1 Study design

Two-sample bidirectional MR analysis was performed to evaluate the causal association between 731 immune cell signatures (seven groups) and HT. In MR analysis, single-nucleotide polymorphisms (SNPs) are used as instrumental variables to estimate the causal impact of exposure variables. Thus, it is essential that the valid instrumental variables (IVs) meet the following three core assumptions (1): IVs are highly associated with exposure (2); IVs must be unrelated to confounders (3); the IVs influence the outcome only via the exposure.

### 2.2 Exposure and outcome data sources

For each immune trait, full genome-wide association study (GWAS) summary statistics are publicly available through the GWAS Catalog server at https://www.ebi.ac.uk/gwas/home (accession number from GCST90001391 to GCST90002121) (15). These GWAS data involve 3,757 subjects of Europeans, which reports the influence of 22 million variants on 731 immune cell signatures.

GWAS summary statistics for HT were acquired from study of Sakaue et al. (16). The data consist 395,640 cases and 379,986 controls of European ancestry. It was not necessary to obtain ethical approval for this study that used publicly available GWAS summary statistics.

### 2.3 Selection of instrumental variables

The threshold of significance of IVs for each immunotype was set to 1 × 10^−5^ according to recent research (15, 17). A threshold value of 0.001 was applied to the linkage disequilibrium parameter (R^2^) in order to select the relevant SNPs. To ensure independence and eliminate the effect of linkage of disequilibrium on the results, a genetic distance of 10,000 kb was set. All of the above operations were performed by using R package “TwoSampleMR”. Furthermore, we conducted a reverse-direction-MR analysis to examine the possibility of reverse-direction causal relationships. For immune cell traits, the significance level was adjusted to 5 × 10^−8^.

### 2.4 Statistical analysis

All analyses were conducted in R (version 4.0.3).

We estimated the causal relationship between immune cell traits and HT using an inverse variance weighted (IVW-random), MR-Egger, and weighted median method and MR pleiotropy residual sum and outlier (MR-PRESSO) test. Among these, the IVW method is the primary approach.

For IVW ratio method MR, the Wald estimator was used to generate MR estimates for each SNP and exposure and outcome associations were both estimated with standard errors based on the Delta method (18). Cochran’s Q statistics were conducted to examine the heterogeneity among estimates between each SNP, and if there is statistical heterogeneity among the findings (P < 0.05), we will select a random-effects model, and otherwise, a fixed-effects model will be used (19). Second, P > 0.05 indicated that there was no horizontal pleiotropy by using the intercept test of MR-Egger (20). In addition, we used the weighted median estimator, which allows the use of invalid instruments when at least half of the instruments are valid in the MR analysis (21). As an additional measure of robustness to the presence of heterogeneity among SNP effects, MR-PRESSO was employed in order to produce an MR estimate (22). As a visual inspection of symmetry and effect estimates, funnel plots and scatter plots were analyzed.

## 3 Result

### 3.1 Exploration of the causal effect of immunophenotypes on HT onset

**Figure 1:**
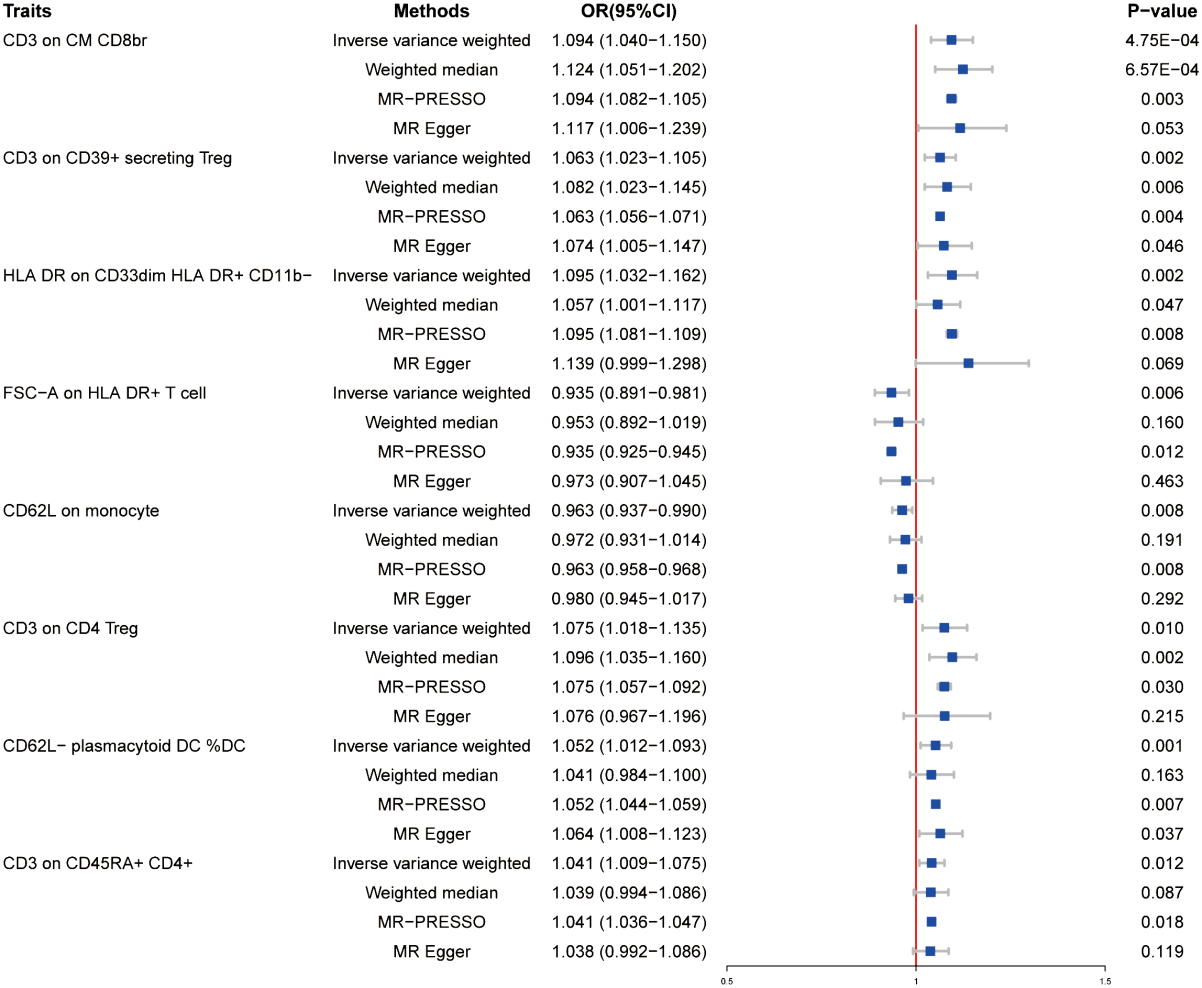
Forest plots showed the causal effects of immune cell traits on HT by using different methods. MR-PRESSO, Mendelian Randomization Pleiotropy Residual Sum and Outlier; OR, adds ratio; CI, confidence interval.

We examined the causal effect of immune cells on HT by using the two-sample MR analysis. When the P-value was set at P < 0.05, a total of 34 immunophenotypes were identified. Of those, top 8 of them are shown in **Figure 1**. As shown in **Figure 1**, the level of CD3 on CD39+ secreting Treg was related to increased susceptibility of HT [odds ratio (OR) = 1.063, 95% confidence interval (CI) = 1.023–1.1015, P = 0.002]. In addition, the level of CD3 on CM CD8br increased the risk of HT (OR = 1.094, 95% CI = 1.040–1.150, P = 0.00048). HLA DR on CD33dim HLA DR+ CD11b− was also observed to be positively associated with the risk of HT (OR = 1.095, 95% CI = 1.032–1.162, P = 0.002). For CD3 on CD4 Treg, a positive association was observed (OR = 1.075, 95% CI = 1.018–1.135, P = 0.01), which was consistent with weight median (OR = 1.096, 95% CI = 1.035–1.160, P = 0.002). The causal effect of CD62L− plasmacytoid DC %DC on HT was estimated to be 1.052 (95% CI = 1.012–1.093, P = 0.001). This association was, however, not supported by the weighted median approach (OR = 1.041, 95% CI = 0.984–1.100, P = 0.163). Additionally, CD3 on CD45RA+ CD4+ was also found to be positively related to the increased risk of HT onset (OR = 1.041, 95% CI = 1.009–1.075, P = 0.012), but the association was not supported by weighted median (OR = 1.039, 95% CI = 0.994–1.086, P = 0.087) and MR Egger (OR = 1.038, 95% CI = 0.992–1.086, P = 0.119). However, the levels of FSC-A on HLA DR+ T cell (OR = 0.935, 95% CI = 0.891–0.981, P = 0.006) and CD62L on monocyte (OR = 0.963, 95% CI = 0.937–0.990, P = 0.008) were associated with disease risk of HT, which suggests the protective role of FSC-A on HLA DR+ T cell and CD62L on monocyte in HT. Details of the MR results and results of heterogeneity and pleiotropy are provided in **Supplementary Table S1**. Detailed information about SNP exceeding the threshold level is listed in **Supplementary Table S2**. We also provided the scatter plots and funnel plots for better demonstration of causality and identification of heterogeneity (**Supplementary Figure 1**).

### 3.2 Exploration of the causal effect of HT on immunophenotypes

**Figure 2:**
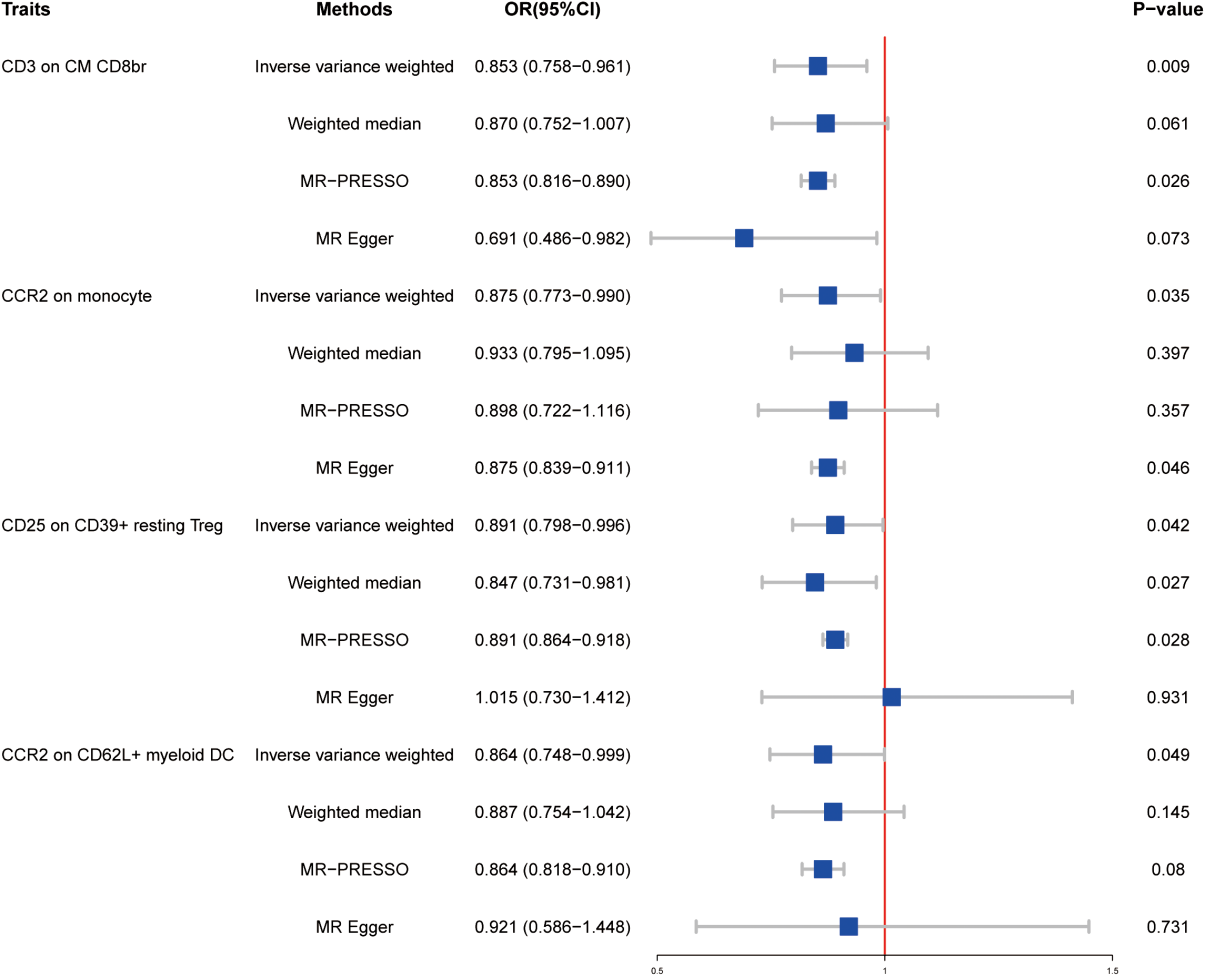
Forest plots showed the causal effects of HT on immunotypes. MR-PRESSO, Mendelian Randomization Pleiotropy Residual Sum and Outlier; OR, adds ratio; CI, confidence interval.

To determine whether the results could be explained by reverse causality, we performed MR analysis in the reverse direction. Four immunophenotypes were detected at P < 0.05: CD3 on CM CD8br (maturation stages of the T-cell panel), CCR2 on monocyte (monocyte panel), CD25 on CD39+ resting Treg (Treg panel), and CCR2 on CD62L+ myeloid DC (cDC panel) (**Figure 2**). Specifically, the OR of HT on CD3 on CM CD8br risk was estimated to be 0.853 (95% CI = 0.758–0.961, P = 0.009) by using the IVW method. A similar result was observed by performing the MR-PRESSO approach (OR = 0.853, 95% CI = 0.816–0.890, P = 0.026). In addition, results from the IVW method revealed that HT onset could decrease the level of CCR2 on monocyte (OR = 0.875, 95% CI = 0.773–0.990, P = 0.035), which was consistent with MR Egger (OR = 0.875, 95% CI = 0.893–0.911, P = 0.046). We also found that the risk of HT was negatively associated with the level of CD25 on CD39+ resting Treg (OR = 0.891, 95% CI = 0.798–0.996, P = 0.042) under the IVW model. A similar association was found for CCR2 on CD62L+ myeloid DC (OR = 0.864, 95% CI = 0.748–0.999, P = 0.049). Detailed results of reverse MR analysis as well as results of heterogeneity and pleiotropy test are shown in **Supplementary Table S3**. Information of SNPs used for these four immune cell traits is listed in **Supplementary Table S4**. Scatter plots and funnel plots also indicated the stability of the results (**Supplementary Figure 2**).

## 4 Discussion

The lymphocytic infiltration in the thyroid gland represented the principal feature of HT; in this way, the thyroid gland can be invaded by lymphocytic cells, which leads to follicular atrophy hyperemia accompanied by oncocytic metaplasia of follicular cells and eventually hypothyroidism (23, 24). Multiple lines of evidence have suggested that immune cells exert function in developments of HT. In a cross-sectional study, the investigators extracted peripheral blood mononuclear cells from both HT patients and healthy controls and the results showed that the percentage of Treg cells was significantly lower in HT patients (8). Another study identified an upregulation of Breg cells but a downregulation of their regulatory activity in HT patients (9, 10). However, whether there is a cause-and-effect relationship is still unclear. 

For the first time, we used the GWAS summary data to analyze the causality between the 731 immune cell traits and HT. To the best of our knowledge, this is the first MR study to explore the causal associations between immunotypes and HT. In this study, among 731 immunotypes, 8 immunotypes had significant causal effects on HT (P < 0.01) and HT was found to have causal effects on four immunotypes (P < 0.05).

As mentioned above, T cells play an essential role in all stages of the development of HT. The CD3 molecule is identified as the T-cell-specific marker which is involved in T-cell development and signaling (25). It is well known that T cells can be divided into two distinct functional subtypes: the CD4+ T helper cells (Th) and the CD8+ cytotoxic T lymphocytes (CTL) (26). According to the difference of surface molecular and endogenous cytokines, CD4+T cells could be classified into several subsets including Th1, Th2, Th9, Th17, Th22, and Tregs (27). Many lines of evidence suggest that the balance between Th17 and Treg cells play a crucial role in progression of HT (28–30). The ratio of Th17/Treg increases as HT progresses, which is consistent with our research findings that the susceptibility of HT was negatively correlated with the level of CD25 on CD39+ resting Treg (31). However, the role of Tregs in occurrence of HT is still less defined. In our study, levels of three types of Tregs (CD3 on CD39+ secreting Treg, CD3 on CD4 Treg, and CD3 on CD45RA+ CD4+) were identified to be positively associated with the risk of HT. Foxp3 is the specific marker of Treg cells (32). It has been shown that genetic polymorphisms of Foxp3 increased the susceptibility of HT in Caucasian women, which could partly explain our results (33). In addition, we found that onset of HT was associated with greater proportions of CD3 on CM CD8br. CD3 on CM CD8br is a type of maturation stage of T cell that derived from bone marrow (34). There is evidence showing that CCR7 mediated the recruitment of mature T cells into the thyroid gland and hence promoted the formation of tertiary structures, which play an important role in HT (35). In addition, our findings reveal that the occurrence of HT decrease the level of CD3 on CM CD8br. This may be attributed to the persistent antigen stimulation leading to T-cell exhaustion in HT (36).

Macrophages are heterogeneous and can differentiate toward various phenotypes under different stimulations (37). Phenotype M1 is pro-inflammatory and can clear necrotic cell debris through releasing pro-inflammatory factors, whereas M2 is an anti-inflammatory phenotype which can facilitate tissue repair and remodeling in the reparative stage of inflammation (38). IL4 is a well-known cytokine that polarizes macrophages to M2 type (39). An animal experiment showed that the ectopic expression of IL4 in thyroid cells increased the incidence of HT and aggravated the severity of HT. Compared with the wild type, mice overexpressing IL4 in the thyroid exhibited more infiltrations of macrophages (40). From this, we speculated that IL4 promoted progression of HT through motivating M2 macrophage polarization. However, our results could not prove that macrophages have a causal relationship with HT, and more studies are needed to prove these aspects.

The dendritic cell (DC) is one of the antigen-presenting cells which could coordinate adaptive immunity and innate immunity (41). Depending on developmental origin, surface marker, and committing transcription factors, DCs can be divided into classical DCs (cDCs), plasmacytoid DCs (pDCs), and monocyte-derived DCs (moDCs) (42). In our study, we found that the risk of HT increased with an increase in the proportion of CD62L− plasmacytoid DC %DC. It was discovered that the density of plasmacytoid dendritic cells was significantly increased in patients with HT compared with controls and the density of plasmacytoid dendritic cells was positively associated with the progression of HT (43). These previous findings are consistent with the result of our present study, indicating that plasmacytoid cells play a critical role in the pathogenesis of HT. Notably, the presence of HT was found to be associated with decreased CCR2 on monocyte and CCR2 on CD62L+ myeloid DC. CCR2 are structurally chemokine receptors which can exert either proinflammatory or anti-inflammatory effects depending on the cellular context (44, 45). To date, there has been limited research on the role of CCR2 in HT. There are reports proving that the concentration of serum CCR2 ligands was higher in healthy controls compared with patients with autoimmune disorders such as multiple sclerosis (46, 47). These findings combined with our results let us to speculate that CCR2 may play an anti-inflammatory role in HT.

HLA DR, an MHC class II cell surface receptor, is encoded by the human leukocyte antigen complex on chromosome 6 region 6P21 (48). Several studies have shown that HLA DR+ thyroid epithelial cells exerted an situ stimulation effect on the immune system within the thyroid gland of patients with HT (49, 50). In our study, we found that HLA DR on CD33dim HLA DR+ CD11b− in the myeloid cell panel was associated with increased HT risk, which suggesting HLA DR+ myeloid cell may play a causative role for HT. However, the effect of FSC-A on the HLA DR+ T cell is opposite to the effect of HLA DR on CD33dim HLA DR+ CD11b− in our study. FSC-A is a morphological parameter; thus, the result suggests that the immune cells morphology might affect their function.

Previous study has identified hub genes with high diagnostic accuracy in HT by the bioinformatic method, and immune profiling revealed that the infiltration of monocyte was inversely associated with the expression of hub genes. This is in agreement with our finding that the risk of HT decreases as the level of CD62L on monocyte increase (51).

TPO is a key enzyme for synthesizing the thyroid hormone (52). It is demonstrated by *in vitro* experiments that anti-TPO could injure thyroid follicular cells through the antibody-dependent cytotoxic mechanism (53). TG is a type of glycoprotein mainly in the thyroid follicular epithelium. A large amount of TG is released when thyroid tissue damage occurs, and the level of anti-TG also increases (54). However, the immunological mechanism underlying the effects of anti-TG in HT is unclear therefore further research is still required.

We have to admit that our research has limitations. First of all, the single population from the Europeans may limit the generalization of our results to other populations. Secondly, due to the lack of individual data such as age or gender, we were unable to perform stratified analysis in this study. Finally, the substantial causal relationship between immune features and HT needs to be further explained due to lack of direct biological evidence.

## 5 Conclusion

In conclusion, this MR study offers genetic evidence for a potential association between immune cell traits and HT. In future studies, animal experiments and large-scale clinical randomized controlled trials are needed to elucidate the underlying mechanism linking these immune cell traits and HT.

## Data availability statement

The original contributions presented in the study are included in the article/**Supplementary material**. Further inquiries can be directed to the corresponding author.

## Ethics statement

Ethical approval was not required for the study involving humans in accordance with the local legislation and institutional requirements. Written informed consent to participate in this study was not required from the participants or the participants’ legal guardians/next of kin in accordance with the national legislation and the institutional requirements.

## Author contributions

ZZ: Conceptualization, Writing – original draft, Writing – review & editing. YG: Writing – review & editing. XP: Data curation, Writing – review & editing. WW: Data curation, Writing – review & editing. HZ: Conceptualization, Writing – original draft, Writing – review & editing.

## Supplementary material

Supplementary Table 1 MR results as well as heterogeneity and pleiotropy results of the causal effect of immune cells on HT (P<0.05).

Supplementary Table 2 Detailed information about SNPs associated with immune cell traits whose P<0.05.

Supplementary Table 3 Results of MR and results of heterogeneity and pleiotropy of causal relationship between HT and immunotypes.

Supplementary Table 4 Detailed information about SNPs of immunotypes which are causally affected by HT.
