# Supplementary material for: Causal role of immune cells in Hashimoto’s thyroiditis: Mendelian randomization study
Source: Front Endocrinol (Lausanne). 2024 May 13;15:1352616. doi: 10.3389/fendo.2024.1352616 (PMC11128540; doi:10.3389/fendo.2024.1352616)
Supplement: Supplementary file 5 [file DataSheet_1.pdf]

Supplementary Material

1.1 Supplementary Figures

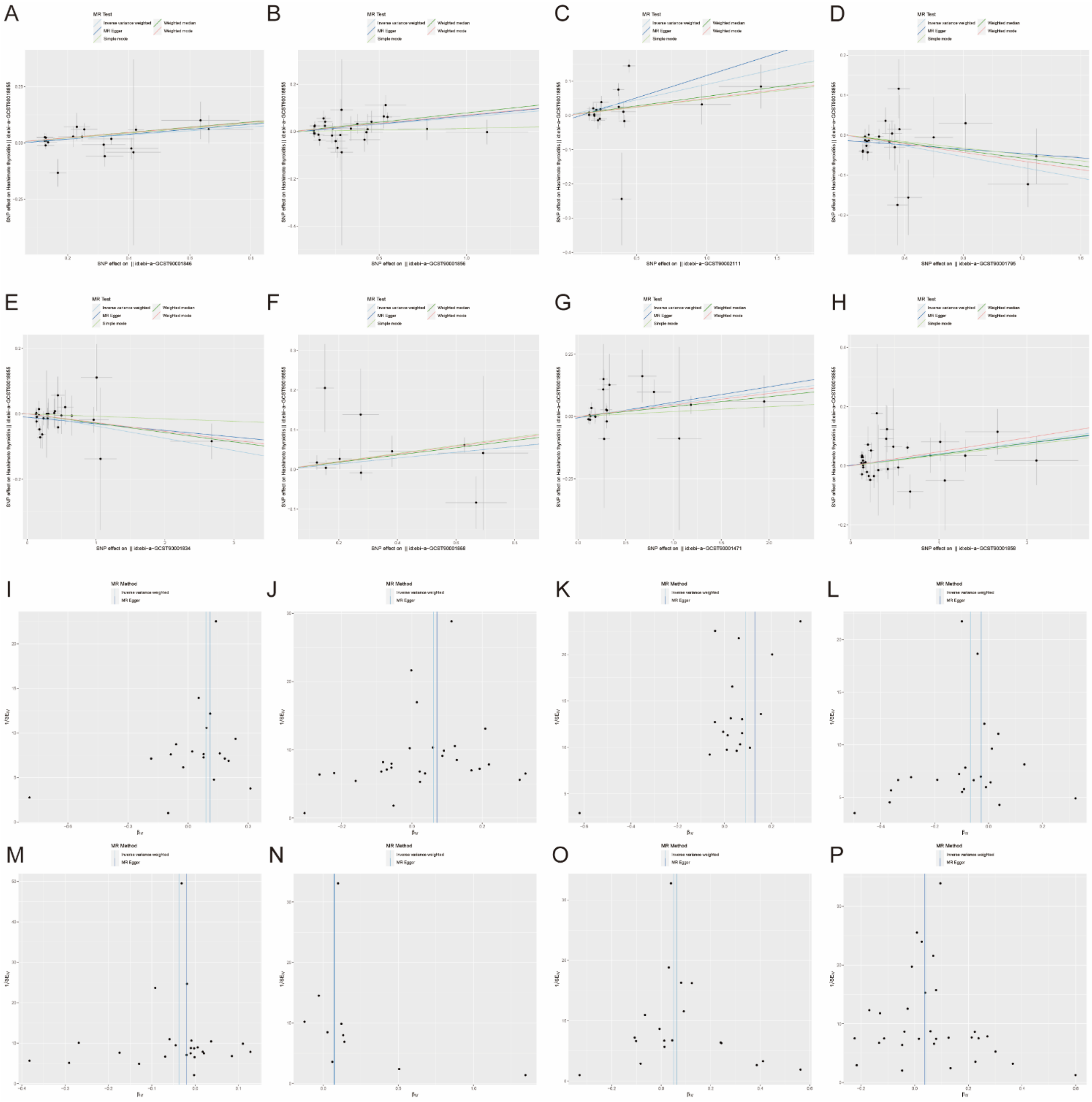

Supplementary Figure 1. Causal effects of immune cell traits on HT. (A) Scatter plot between CD3 on CM CD8br and HT risk; (B) Scatter plot between CD3 on CD39+ secreting Treg and HT risk; (C) Scatter plot between HLA DR on CD33dim HLA DR+ CD11b- and HT risk; (D) Scatter plot between FSC-A on HLA DR+ T cell and HT risk; (E) Scatter plot between CD62L on monocyte and HT risk; (F) Scatter plot between CD3 on CD4 Treg and HT risk; (G) Scatter plot between CD62L-

plasmacytoid DC %DC and HT risk; (H) Funnel plot between CD3 on CD45RA+ CD4+ and HT risk; (I) Funnel plot between CD3 on CM CD8br and HT risk; (J) Funnel plot between CD3 on CD39+ secreting Treg and HT risk; (K) Funnel plot between HLA DR on CD33dim HLA DR+ CD11b- and HT risk; (L) Funnel plot between FSC-A on HLA DR+ T cell and HT risk; (M) Funnel plot between CD62L on monocyte and HT risk; (N) Funnel plot between CD3 on CD4 Treg and HT risk; (O) Funnel plot between CD62L- plasmacytoid DC %DC and HT risk; (P) Funnel plot between CD3 on CD45RA+ CD4+ and HT risk.

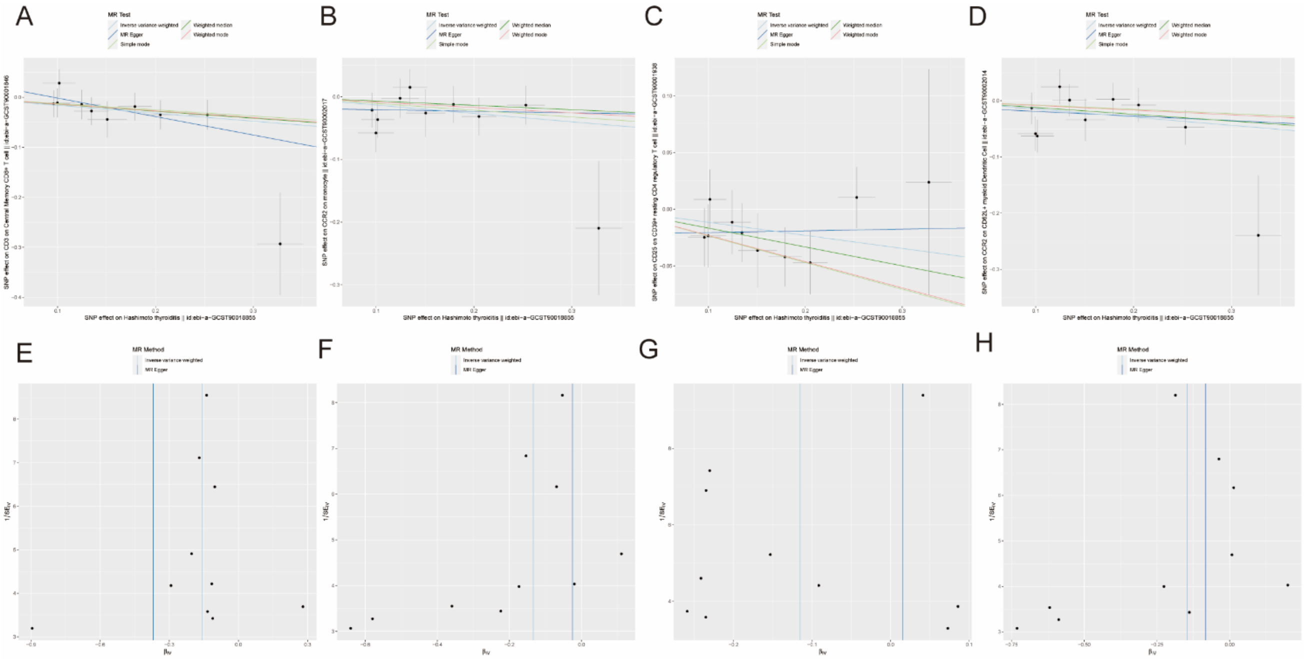

Supplementary Figure 2. Causal effects of HT on immune cell traits. (A) Scatter plot between CD3 on CM CD8br and HT risk; (B) Scatter plot between CCR2 on monocyte and HT risk; (C) Scatter plot between HLA DR on CD25 on CD39+ resting Treg and HT risk; (D) Scatter plot between CCR2 on CD62L+ myeloid DC and HT risk; (E) Funnel plot between CD3 on CM CD8br and HT risk; (F) Funnel plot between CCR2 on monocyte and HT risk; (G) Funnel plot between HLA DR on CD25 on CD39+ resting Treg and HT risk; (H) Funnel plot between CCR2 on CD62L+ myeloid DC and HT risk.
